# Unlocking the Potential of *vB*_*EfaS*_*LOK1*: A Newly Isolated Bacteriophage Against *Enterococcus faecalis*

**DOI:** 10.3390/microorganisms13102414

**Published:** 2025-10-21

**Authors:** Laura Marcela Plata Suarez, Salvador Del Valle Balbuena, Isamar Leticia Becerra Mejía, Alejandra Aidee Loera Piedra, Cristina Domínguez Espinoza, Arantxa Monserrat Ángeles González, Araceli Contreras Rodríguez, Alejandra Aquino Andrade, Sergio Francisco Martínez Díaz, Ma. Guadalupe Aguilera Arreola

**Affiliations:** 1 Medical Bacteriology Laboratory, National School of Biological Sciences, Instituto Politécnico Nacional, Mexico City 11340, Mexico; lplatas2200@alumno.ipn.mx (L.M.P.S.); ibecerram1000@alumno.ipn.mx (I.L.B.M.); aloerap2000@alumno.ipn.mx (A.A.L.P.); cdomingueze1600@alumno.ipn.mx (C.D.E.); aangeles2401@alumno.ipn.mx (A.M.Á.G.); 2 Soil and Water Laboratory, Department of Biotechnology Engineering, Polytechnic University of the State of Morelos (UPEMOR), Jiutepec Morelos 62574, Mexico; 22070077@upermor.edu.mx; 3 General Microbiology Laboratory, National School of Biological Sciences, Instituto Politécnico Nacional, Mexico City 11340, Mexico; acontreras@ipn.mx; 4 Molecular Microbiology Laboratory, Instituto Nacional de Pediatría, Mexico City 04530, Mexico; aaquinoa@pediatria.gob.mx; 5 Microbiology and Molecular Biology Laboratory, Interdisciplinary Center for Marine Sciences, Instituto Politécnico Nacional, La Paz 23096, Mexico

**Keywords:** *Enterococcus faecalis* phage, phage therapy, bacteriophages, antibiotic resistance, whole genome sequencing, phylogenomic analysis

## Abstract

Phage therapy has emerged as a promising alternative for combating infections caused by drug-resistant pathogens. Among these, *Enterococcus faecalis* remains a significant public health concern due to its persistence in clinical settings and frequent involvement in healthcare-associated infections (HAIs). In this study, we report the characterization of the lytic bacteriophage *vB_EfaS_LOK1*, isolated from urban sewage using *E. faecalis* strain IIH-74.4 as the host. Transmission electron microscopy revealed morphological features consistent with the phages formerly classified within the *Siphoviridae* family. The phage exhibited high thermal and pH stability, remaining viable up to 70 °C and within a pH range of 4–11. It displayed a latent period of 20 min and a burst size of 72 PFU/cell. Notably, *vB_EfaS_LOK1* exhibited a narrow host range, lysing only the strain used for their isolation. Genomic analysis revealed a 41.2 kb double-stranded DNA genome devoid of known virulence or antibiotic resistance genes. Phylogenomic analysis classified the phage within the genus *Efquatrovirus* (Caudoviricetes), suggesting it represents a newly isolated bacteriophage species. Functional annotation identified genes related to DNA replication, host interaction, and bacterial lysis, including endolysins and holins with putative antimicrobial properties. Long-term stability assays demonstrated that tryptic soy broth (TSB) with CaCl_2_/MgCl_2_ at 4 °C maintained viability for at least 90 days. Collectively, these findings support the potential of *vB_EfaS_LOK1* as a potential candidate for the development of phage-based therapies targeting *E. faecalis*.

## 1. Introduction

*Enterococcus faecalis* is a Gram-positive, cocci-shaped, facultatively anaerobic, non-spore-forming bacterium that constitutes part of the normal microbiota of the human gastrointestinal tract, oral cavity, and genitourinary system. Withing these niches, *E. faecalis* contributes to host homeostasis by preventing the colonization by pathogenic bacteria, supporting nutrient metabolism, and modulating immune responses [1,2]. However, it is an opportunistic pathogen and is frequently implicated in a wide range of infections, including catheter-associated urinary tract infections (CAUTIs), endocarditis, peritonitis, colitis, diabetic foot ulcers, and surgical site infections, particularly in healthcare settings [3]. Remarkably, *E. faecalis* was responsible for 64.7% of healthcare-associated enterococcal infections (HAIs) reported worldwide between 1997 and 2016 [4,5,6].

The pathogenic potential of *E. faecalis* is attributed to a diverse repertoire of virulence factors, including its capacity to form biofilms, invade host tissues, evade phagocytic clearance, and modulate immune response [7,8]. This species also shows remarkable tolerance to harsh environmental conditions, including alkaline pH, bile salts, high sodium concentrations, detergents, and desiccation [9]. Moreover, *E. faecalis* exhibits both intrinsic and acquired resistance to antimicrobials, which significantly complicates treatment efforts. It is intrinsically resistant to cephalosporins, trimethoprim–sulfamethoxazole, and aminoglycosides and displays variable resistance to penicillin, ampicillin, and high-level aminoglycosides resistance (HLAR) phenotypes [8,10]. Alarmingly, it can also acquire resistance to last-line antibiotics such as vancomycin mainly through horizontal gene transfer of *vanA* or *vanB* clusters [11].

The global rise in multidrug-resistant bacterial infections has renewed scientific and clinical interest in bacteriophage therapy as a targeted and potentially effective alternative to conventional antibiotics [12]. Bacteriophages, or phages, are viruses that specifically infect bacteria by binding to host surface receptors and are considered the most abundant biological entities on Earth, with an estimated population of 10^31^ particles [13]. Phage therapy offers notable advantages over broad-spectrum antibiotics, including host specificity that minimizes disruption to the commensal microbiota, self-amplification at the infection site, low inherent toxicity, and the ability to disrupt established biofilms, which are frequently implicated in persistent infections. Furthermore, phages can be administered in combination with antibiotics to improve antimicrobial efficacy and reduce resistance, as well as in cocktails to broaden the host range and enhance treatment outcomes [14,15,16,17,18]. Despite these benefits, the clinical implementation of phage therapy remains limited by the narrow host range of most phages, limited high-quality clinical evidence, and the lack of regionally tailored phage libraries to support personalized therapeutic approaches [19,20,21,22].

This study aimed to isolate and characterize the lytic phage *vB_EfaS_LOK1*, which targets a clinical strain of *E. faecalis* with the objective of contributing to the development of phage-based therapeutic strategies.

## 2. Materials and Methods

### 2.1. Phage Isolation

Bacteriophages were isolated from urban sewage obtained from a wastewater treatment plant in Mexico City, following standard protocols with minor modifications [23,24,25,26,27]. The clinical *Enterococcus faecalis* strain IIH-74.4 was used as the host for phage enrichment. Sewage samples were sonicated in a bath sonicator (Branson Ultrasonics, model 5510, Danbury, CT, USA) at 40 kHz for 30 s to 4 min at 4 °C, centrifuged at 13,000× *g* for 10 min at 4 °C, and filtered through 0.22 μm pore-size membranes (Corning, Kaiserslautern, Germany). A 3 mL aliquot of the filtrate was mixed with 100 μL of a mid-log phase *E. faecalis* IIH-74.4 culture (6 × 10^8^ CFU/mL) in 3 mL of 2x supplemented TSB and incubated at 37 °C for 24 h at 150 rpm. After incubation, 1% chloroform (Sigma-Aldrich, St. Louis, MO, USA) was added, and the culture was centrifuged at 15,000× *g* for 10 min at 4 °C. The supernatant was then filtered through sterile 0.22 μm membranes to recover the phage containing lysate.

### 2.2. Phage Purification and Lysate Selection

Phage presence assessment was carried out using the double-layer agar method as previously described [28,29]. After overnight incubation at 37 °C, individual plaques were selected and inoculated into fresh flasks containing a mid-log phase culture of *E. faecalis* IIH-74.4. Cultures were incubated at 37 °C until complete lysis was observed. The resulting lysates were treated with 1% (*v*/*v*) chloroform, centrifuged at 15,000× *g* for 10 min at 4 °C, and filtered through sterile 0.22 μm membranes. The filtrates were diluted in TSB and replated onto host strain lawns using the double-layer agar method. The single plaque purification process was repeated four times to ensure clonal purity. For this study, a crude lysate that consistently produces complete lysis (named LOK1 and the corresponding phage as *vB_EfaS_LOK1*) was selected for further analysis. Phage titers were determined as plaque-forming units per milliliter (PFU/mL), and final lysates were stored at 4 °C until use.

### 2.3. Microscopic Analysis of Phage Morphology

Transmission electron microscopy (TEM) was performed to examine the morphology of *vB_EfaS_LOK1* virions at the Electron Microscopy Unit of the *Instituto de Biotecnología, Universidad Nacional Autónoma de México* (UNAM), Cuernavaca, Mexico. Phage lysates were concentrated by overnight incubation with 10% polyethylene glycol 8000 (PEG8000; Sigma-Aldrich, Merck KGaA, Darmstadt, Germany) at 4 °C. Phage particles were pelleted by centrifugation at 9000× *g* for 15 min at 4 °C and resuspended in 2 mL of SM buffer (5.8 g NaCl, 1.2 g MgSO_4_, and 50 mL 1 M Tris-HCl, pH 7.5). Virions were purified by cesium chloride (CsCl) density gradient centrifugation, following a previously described method [30], with minor modifications. Gradients were prepared using CsCl solutions with densities of 1.3, 1.4, and 1.6 g/mL. The phage suspension was centrifuged at 79,000× *g* for 2 h at 4 °C, and the visible phage band was collected immediately. Virions were negatively stained with 2% uranyl acetate (pH 7.0) for 90 s and visualized using a Zeiss Libra 120 transmission electron microscope (Carl Zeiss AG, Oberkochen, Germany) operated at 80 kV.

### 2.4. Host Range Assay

The host range of *vB_EfaS_LOK1* phage was evaluated by spot testing. Briefly, 10 μL of phage suspension was spotted onto bacterial lawns of different strains grown on TSA or LB agar supplemented with MgCl_2_ (222 mg/L, 2.3 mM) and CaCl_2_ (222 mg/L, 2.0 mM). Plates were incubated at 37 °C for 24 h, and susceptibility to *vB_EfaS_LOK1* was determined by the presence of clear lysis zones at the inoculation sites. Results were recorded as positive (+) when clear zones were observed, or negative (−) when no lysis occurred [30,31]. A total of 30 clinical isolates were used for host range testing. This panel included 19 strains of *Enterococcus* (eleven *E. faecium* and eight *E. faecalis*), along with clinical isolates of other bacterial species: *Staphylococcus aureus* (n = 5), *Acinetobacter baumannii* (n = 2), and one isolate each of *Klebsiella pneumoniae*, *Pseudomonas aeruginosa*, *Escherichia coli*, and *Enterobacter cloacae* (Table S1, Supplementary Materials). All bacterial strains were clinical isolates obtained from patients at *Centro Médico Nacional “20 de Noviembre”, Hospital Infantil de México “Federico Gómez” (HIMFG)*, and the *Instituto Nacional de Pediatría (INP)* in Mexico City.

### 2.5. One-Step Growth Curve

A one-step growth curve was performed following the method reported by Liang et al. [32]. A mid-exponential phase culture of *E. faecalis* IIH-74.4 (1.5 × 10^8^ CFU/mL) was infected with phage *vB_EfaS_LOK1* at a multiplicity of infection (MOI) of 10 and incubated at room temperature for 6 min to allow adsorption. The mixture was centrifuged at 15,000× *g* for 10 min at 4 °C, and the pellet was resuspended in 10 mL of TSB supplemented with MgCl_2_ (222 mg/L, 2.3 mM) and CaCl_2_ (222 mg/L, 2.0 mM). The phage–host suspension was incubated at 37 °C, and aliquots were collected every 10 min. Each sample was centrifuged, serially diluted, and titrated using the double-layer agar method to determine the phage concentration over time.

### 2.6. Thermal and pH Stability

The thermal and pH stability of phage *vB_EfaS_LOK1* was assessed following a previously described protocol [33], with minor modifications. Phage suspensions (1 × 10^8^ PFU/mL) prepared in TSB supplemented with MgCl_2_ (222 mg/L, 2.3 mM) and CaCl_2_ (222 mg/L, 2.0 mM) were incubated at different temperatures (4, 25, 37, 50, 60, 70, and 80 °C) for 1 h. For pH stability, phage suspensions were incubated at 25 °C in buffer solutions adjusted to pH 3, 4, 5, 7, 9, 11, 12, and 13 at 25 °C for 1 h. After incubation, residual phage titers were determined using the double-layer agar method.

### 2.7. Evaluation of Storage Conditions

Phage storage stability was evaluated using suspensions prepared in either TSB supplemented with MgCl_2_ (222 mg/L, 2.3 mM) and CaCl_2_ (222 mg/L, 2.0 mM) with a pH of 7.2, or SM buffer (pH 7.5). For storage at −20 °C, 15% (*v*/*v*) glycerol was added. Phage stocks were stored at 4 °C and −20 °C at an initial concentration of 1 × 10^11^ PFU/mL. Phage titers were determined using the double-layer agar method after 8, 30, and 90 days of storage [34].

### 2.8. Phage DNA Isolation, Genome Sequencing, Assembly, and Annotation

A 1 mL suspension of phage *vB_EfaS_LOK1* at a concentration of 1 × 10^10^ PFU/mL was filtered through a sterile 0.22 µm pore-size membrane to remove bacterial debris. The filtrate was treated with DNase I (20 mg/mL: Sigma-Aldrich, Cat. D5319) and RNase A (10 mg/mL; Sigma-Aldrich, Cat. R4875) at 37 °C for 30 min to degrade contaminating host nucleic acids. Enzymes were inactivated by incubation at 75 °C for 5 min. Genomic DNA was subsequently extracted using the Phage DNA Isolation Kit (Norgen Biotek, Thorold, ON, Canada, Cat. 46800) according to the manufacturer’s instructions. DNA quality and integrity were assessed using an Agilent 5400 Fragment Analyzer (Agilent, Santa Clara, CA, USA). Sequencing libraries were prepared following standardized protocols for viral DNA and sequenced on the Illumina NovaSeq 6000 platform, generating 150 bp paired end reads.

All subsequent bioinformatic analyses were conducted on the Galaxy platform 25.0.4.dev0 European server (https://usegalaxy.eu/about, accessed on 14 October 2025) [35]. Raw reads were trimmed using Trimmomatic v0.39, with a 15 bp sliding window to remove low quality bases and filtering out reads shorter than 30 bp or with Phred scores below 27 [36]. High-quality reads were assembled de novo using SPAdes v3.15.4 with error correction enabled [37]. Assembly quality and topology were determined by mapping raw reads to the assembled contig with Bowtie2: 2.5.4+galaxy0, and termini were identified using PhageTerm 1.0.8 cross-validated with CheckV 1.0.3 [38,39].

Gene prediction and annotation were performed using Pharokka v3.0.0 [40] in combination with Phanotate v1.5.0 for accurate open reading frame detection. Additional functional annotation was conducted using Abricate v1.0.1 [41] querying the ResFinder v4.1 [42] and the Virulence Factor Database (VFDB) [43] databases. The complete annotated genome of *vB_EfaS_LOK1* was deposited in GenBank with the accession number PV780768.

The lifestyle of *vB_EfaS_LOK1* was predicted using the Phage.AI platform (PhageAI S.A., 2023; https://www.phage.ai (accessed on 2 June 2025), a deep learning-based platform trained on curated phage genomes. The complete genome sequence was converted into k-mer vectors (k = 6) to capture specific sequence patterns, which were subsequently reduced in dimensionality using Uniform Manifold Approximation and Projection (UMAP) to facilitate clustering with reference phages. A supervised classifier trained on annotated datasets was then applied to predict the lifestyle (virulent or temperate) based on the embedded sequence features.

### 2.9. Phylogenomic Analysis

The assembled genome of phage *vB_EfaS_LOK1* was subjected to a multi-step phylogenomic analysis. Initially, the complete FASTA sequence was queried against the NCBI nucleotide database using BLASTn v2.12.0 [44] to identify the closest relatives. The comparative dataset was curated to include (i) the 14 ICTV-recognized type species of *Efquatrovirus*; (ii) the closest BLASTn match, *vB_OCPT_CCS4*; (iii) five additional high-scoring BLASTn relatives; and (iv) an external outgroup, *Staphylococcus phage Twort* (complete genome).

Average Nucleotide Identity (ANI) values were calculated using the OAT OrthoANI v0.93.1 on the EzBioCloud platform, which implements the OrthoANI algorithm [45] to refine estimates of genomic relatedness and assess coding sequence conservation. In addition, intergenomic similarities were assessed with VIRIDIC v1.1 [46], which applies ICTV-recommended metrics for species-level demarcation. For genome-based phylogenetic inference, the Virus Classification and Tree Building Online Resource (VICTOR) platform [47] was used, applying the Genome-BLAST Distance Phylogeny (GBDP) method with the dDDH6 formula to compute genome-to-genome distances to construct an accurate phylogenomic tree.

### 2.10. Statistical Analysis

Phage stability under different pH and temperature conditions, was analyzed using one-way analysis of variance (ANOVA) followed by Tukey’s post hoc test for multiple comparisons. For the evaluation of storage stability, where comparisons were made between each time point to the baseline (day 0) within the same condition, a two-way ANOVA followed by Dunnett’s multiple comparisons test was applied. A *p*-value ≤ 0.05 was considered statistically significant. All statistical analyses were performed using GraphPad Prism v9.4.1 for Windows (GraphPad Software, 2022; San Diego, CA, USA; https://www.graphpad.com (accessed on 10 June 2025).

## 3. Results

### 3.1. Morphology and Host Range of Bacteriophage vB_EfaS_LOK1

**Figure 1:**
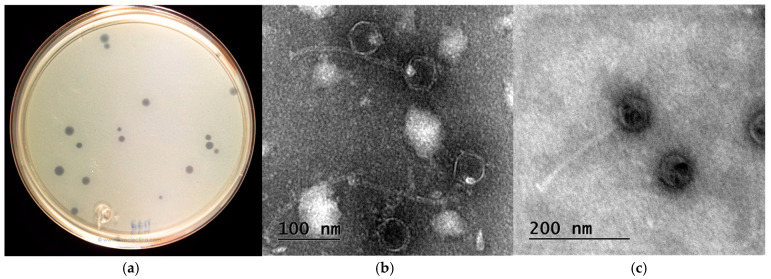
(**a**) Plaque morphologies of phage *vB_EfaS_LOK1*, and (**b**,**c**) transmission electron microscope images of *vB_EfaS_LOK1*.

Bacteriophage *vB_EfaS_LOK1* was isolated from municipal wastewater using the double-layer agar method. Plaque assays revealed clear, round plaques measuring approximately 2–4 mm in diameter, each surrounded by a diffuse halo of ~1 mm (Figure 1a). These morphological features are indicative of a lytic replication cycle, which was further confirmed by observations in liquid culture. TEM showed that *vB_EfaS_LOK1* possesses an icosahedral capsid and a long, flexible, noncontractile tail. Capsid diameter ranged from 48 to 55 nm, and tail length from 184 to 205 nm (Figure 1b,c), consistent with the morphological characteristics of phages formerly classified within the *Siphoviridae* family.

The host range of *vB_EfaS_LOK1* was assessed by spot testing against a panel of 30 clinical isolates and revealed a narrow spectrum pf activity. Lytic activity was observed exclusively against the original *E. faecalis* strain, with no detectable infectivity toward other *Enterococcus* strains or any of the non-enterococcal species tested.

### 3.2. One-Step Growth Curve

**Figure 2:**
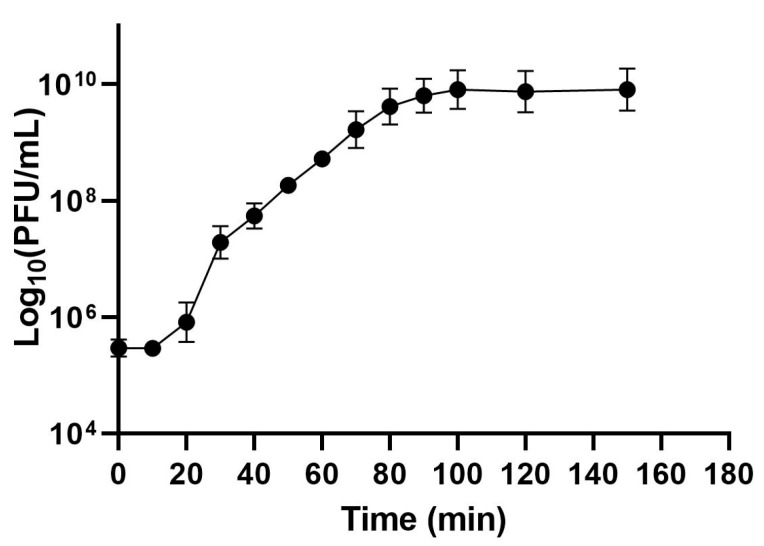
One-step growth curve of phage *vB_EfaS_LOK1*. *E. faecalis* host strain was infected at a multiplicity of infection (MOI) of 10. Phage titer was determined using the double-layer plaque assay. Data are expressed as the mean ± SEM of three independent experiments.

The replication dynamics of phage *vB_EfaS_LOK1* were evaluated through a one-step growth assay. The latent period, defined as the time between adsorption and the onset of the first burst, was approximately 20 min. The subsequent rise period extended to approximately 70 min. The average burst size was estimated as ~72 virions per infected cell, calculated by dividing the phage titer at the plateau phase by the initial number of host bacterial cells (Figure 2). These results indicate that phage *vB_EfaS_LOK1* possesses a relatively short latent period, a prolonged rise phase, and a moderate burst size, characteristics consistent with effective lytic activity.

### 3.3. Thermal and pH Stability

**Figure 3:**
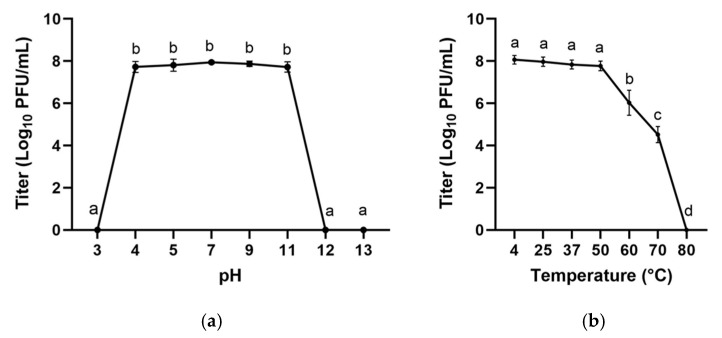
Stability of phage *vB_EfaS_LOK1.* Data are expressed as the mean ± SD of three independent experiments. Different letters represent significant differences (*p* ≤ 0.05) based on ANOVA and Tukey tests. (**a**) pH tolerance. (**b**) Thermal stability.

The stability of phage *vB_EfaS_LOK1* was assessed under different pH and temperature conditions. As shown in Figure 3a, the phage maintained high viability within a broad pH range of 4 to 11, with no statistically significant differences in titer observed within this interval (*p* > 0.05), as indicated by the shared statistical groupings. In contrast, the phage was completely inactivated under strongly acidic (pH 3) and highly alkaline conditions (pH 12 and 13), indicating that *vB_EfaS_LOK1* is sensitive to extreme pH environments.

Thermal tolerance of phage *vB_EfaS_LOK1* was assessed across a temperature gradient (Figure 3b). The phage remained stable from 4 °C to 37 °C, with titers consistently above 8.0 log_10_ PFU/mL and no statistically significant differences observed within this range (*p* > 0.05). However, a significant reduction in viability was detected at 60 °C and 70 °C, and complete inactivation occurred at 80 °C (*p* ≤ 0.05). These results indicate that *vB_EfaS_LOK1* is thermally stable under standard and moderate elevated temperatures but is susceptible to heat-induced inactivation at high temperatures.

### 3.4. Evaluation of Storage Conditions

The long-term stability of phage *vB_EfaS_LOK1* was evaluated over a 90-day period under various storage conditions. When stored in SM buffer at either 4 °C or −20 °C, phage titers decreased by approximately 2 to 4 log_10_ units. In contrast, storage in TSB supplemented with CaCl_2_ and MgCl_2_ resulted in significantly better preservation, with titer reductions limited to approximately 1 log_10_ unit, particularly in the samples maintained at 4 °C (*p* ≤ 0.05).

By day 8, phage titers in SM buffer at 4 °C remained comparable to those in TSB stored at −20 °C. However, at days 30 and 90, phages stored in SM buffer -particularly under freezing conditions- exhibited a marked loss in viability, like that observed in SM buffer with glycerol at −20 °C. Only the samples stored in TSB supplemented with MgCl_2_ and CaCl_2_ at 4 °C maintained stable titers throughout the 90 days period, whereas all other conditions showed a statistically significant reduction in titer starting at day 8.

**Figure 4:**
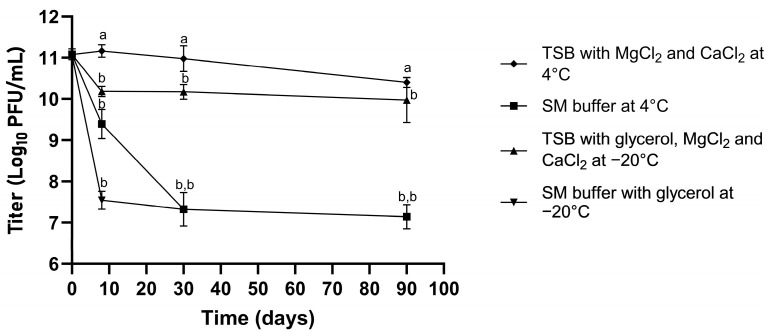
Stability of phage *vB_EfaS_LOK1* under different storage conditions. Phage titers (Log10 PFU/mL) were monitored over time in different storage media: TSB: tryptic soy broth and SM buffer: Sodium–Magnesium buffer. Data are presented as means ± standard deviation (n = 3). Statistical comparisons were performed using Dunnett’s post hoc test was used to determine significant differences between each time point and day 0 within the same storage condition. Data points labeled with different letters indicate statistically significant differences from day 0 (*p* ≤ 0.05). Specifically, points sharing the letter “a” are not significantly different from day 0, while those labeled with “b” are significantly different from their corresponding day 0 value.

Overall, TSB supplemented with CaCl_2_ and MgCl_2_ at 4 °C probed to be the most effective condition for preserving phage viability (Figure 4).

### 3.5. Genomic Characterization and Functional Analysis

The complete genome of phage *vB_EfaS_LOK1* was subjected to comprehensive functional and lifestyle analysis to evaluate its genetic composition, biological functionality, and therapeutic potential. Lifestyle prediction using the Phage.AI platform classified *vB_EfaS_LOK1* as virulent, consistent with a strictly lytic replication strategy. Additionally, genome screening with Abricate against the ResFinder and VFDBs confirmed the absence of antimicrobial resistance genes and known bacterial virulence factors, reinforcing its biosafety and suitability for therapeutic applications.

**Figure 5:**
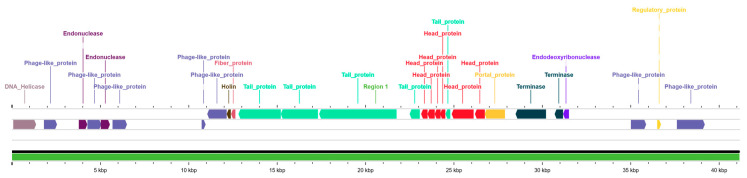
Genomic organization of *vB_EfaS_LOK1*. The genomic map of phage *vB_EfaS_LOK1* (41,176 bp) displays the linear arrangement of genes involved in key functional modules. Only genes with predicted or assigned functions are shown, including those encoding structural proteins, replication enzymes, lysis factors and other functional elements. Distinct color-coded blocks represent different genes, facilitating visualization of the genome’s modular organization and functional architecture.

The genome of *vB_EfaS_LOK1* consists of a linear double-stranded DNA molecule of 41,176 bp with a GC content of 34.80%, and its termini were identified as cohesive ends based on PhageTerm analysis (Supplementary Materials). Structural and functional annotation identified the major genetic modules expected in a lytic phage (Figure 5). These included genes encoding structural proteins, such as major and minor capsid components, tail proteins, head–tail adaptors, and tail terminators, DNA packaging genes, including the large and small subunits of the terminase complex, and replication-associated enzymes, including DNA polymerase, helicase, and primase.

Furthermore, lysis-related genes such as endolysin and holins were detected, suggesting the phage’s potential to degrade the bacterial cell wall and promote progeny release. Several HNH endonucleases were annotated, potentially representing mobile genetic elements rather than conserved functional determinants; their precise roles remain to be elucidated. Collectively, these genomic features support the classification of *vB_EfaS_LOK1* as a safe and promising candidate for phage therapy targeting multidrug-resistant *E. faecalis*.

### 3.6. Phylogenomic Analysis and Evolutionary Relationships

**Figure 6:**
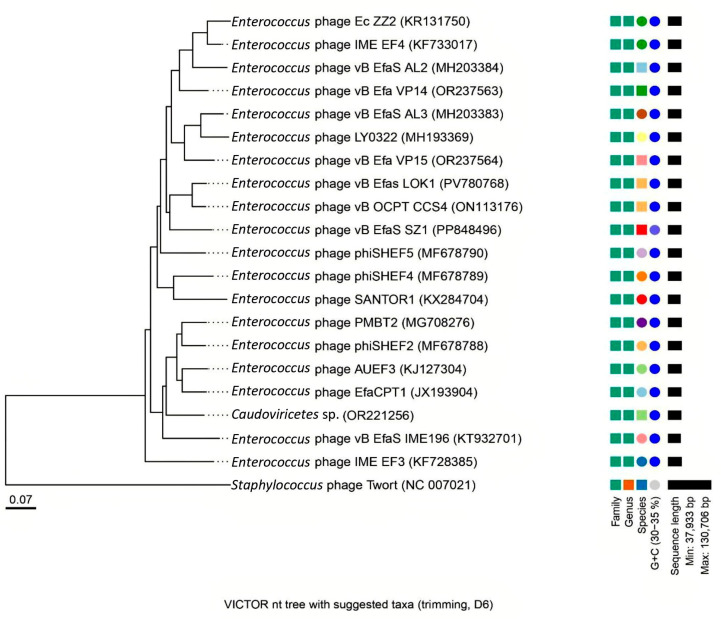
Phylogenomic classification of phage *vB_EfaS_LOK1*. A phylogenomic tree was generated using the Virus Classification and Tree Building Online Resource (VICTOR) based on Genome-BLAST Distance Phylogeny (GBDP) and dDDH6 values. The analysis included phage *vB_EfaS_LOK1*, the 14 ICTV-recognized type species of *Efquatrovirus*, the closest BLASTn relative *vB_OCPT_CCS4*, and additional enterococcal phages identified by BLASTn. *Staphylococcus phage Twort* served as an external outgroup to root the tree. Branch support values represent bootstrap frequencies, and branch lengths are proportional to intergenomic distances. *vB_EfaS_LOK1* clustered within the genus *Efquatrovirus* and was most closely related to *vB_OCPT_CCS4*, sharing 96.64% Average Nucleotide Identity (ANI). Together, these phages constitute a distinct species-level lineage, differentiated from all ICTV-recognized type species, supporting their classification as a newly isolated species within the genus *Efquatrovirus*.

The phylogenomic tree generated using the Genome-BLAST Distance Phylogeny (GBDP) method on the VICTOR platform revealed the evolutionary relationships of phage *vB_EfaS_LOK1* within the broader context of bacteriophage diversity (Figure 6). Phage vB_EfaS_LOK1 clustered within the genus *Efquatrovirus* (class *Caudoviricetes*), consistent with other members of this phylogenomic group. *Enterococcal* phages assigned to this lineage generally exhibited conserved genomic attributes, including genome sizes of approximately 40 kb, GC contents between 34.5% and 34.8%, and linear genomes with cohesive ends; *vB_EfaS_LOK1* conformed to this conserved profile, further supporting its assignment to *Efquatrovirus*.

**Figure 7:**
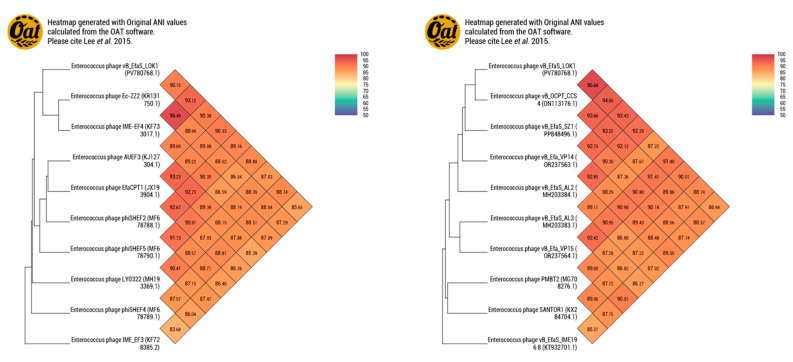
ANI values were calculated using the Orthologous Average Nucleotide Identity Tool (OAT) on the EzBioCloud platform. The dataset comprised phage *vB_EfaS_LOK1*, the 14 ICTV-recognized type species of the genus *Efquatrovirus*, its closest BLASTn relative *vB_OCPT_CCS4*, and additional *Enterococcus* phages. The heatmap shows that *vB_EfaS_LOK1* shares 96.64% ANI with *vB_OCPT_CCS4*, supporting their assignment to the same species-level cluster. In contrast, ANI values between this pair and all ICTV type species fall below the 95% threshold, corroborating their classification as a new species within *Efquatrovirus* [45].

Average Nucleotide Identity (ANI) analysis using the OAT (EzBioCloud) revealed that *vB_EfaS_LOK1* shares 96.64% ANI with its closest relative, *vB_OCPT_CCS4*, consistent with their assignment to the same species-level cluster. In contrast, ANI values between those phages and the 14 ICTV-recognized type species of *Efquatrovirus* were all below the 95% threshold for species demarcation. Intergenomic similarity values calculated with VIRIDIC (Figure S1, Supplementary Materials) corroborated these results. The ANI heatmap (Figure 7) further illustrates these relationships, showing that *vB_EfaS_LOK1* and *vB_OCPT_CCS4* constitute a distinct species-level cluster separate from all ICTV type species, thereby supporting their classification as a newly isolated bacteriophage within the genus *Efquatrovirus*.

## 4. Discussion

In recent decades, the widespread and often indiscriminate use of antibiotics, coupled with bacterial mutations driven by selective pressure, has significantly accelerated the emergence of antibiotic resistance among bacterial populations [48,49]. The ongoing rise in antibiotic resistance poses serious public health challenges, including prolonged illness, higher rates of complication, increased reliance on more toxic or expensive treatments, and higher mortality rates [50]. *Enterococcus faecalis* is considered one of the most concerning pathogens due to its prominent role in HAIs and the high prevalence of multidrug-resistant (MDR) strains [51].

Phage therapy has emerged as a promising alternative for combating infections caused by MDR bacteria, including *E. faecalis* [52]. The identification of multiple *E. faecalis*-specific phages has facilitated the development of phage cocktails aimed at minimizing the emergence of resistance. However, the effectiveness of phage therapy is often influenced by biogeographical variation among bacterial populations. Strains can differ considerably across geographic regions, implying that phages isolated in one location may not exhibit the same efficacy against strains originating from another [53,54,55]. This highlights the need for ongoing isolation and characterization of bacteriophages specifically adapted to regionally prevalent pathogenic strains [54]. In this study, we isolated the strictly lytic bacteriophage *vB_EfaS_LOK1* from urban sewage using an MDR *E. faecalis* host strain and conducted comprehensive functional and genomic characterization.

Phage *vB_EfaS_LOK1* produced clear, lytic plaques measuring 2–4 mm in diameter, each surrounded by a faint halo. Unlike most reports where plaque morphology tends to stabilize after rounds of purification, *vB_EfaS_LOK1* exhibited notable size heterogeneity even after five purification cycles. While uniform plaque sizes of approximately 2–3 mm are commonly reported for *E. faecalis* phages [29], similar variability has been observed in other cases. For instance, although phage phiNASRA1 was reported to form ~3 mm plaques, Figure 1 of the same study reveals a range of plaque sizes [56]. A comparable phenomenon has been reported in phages infecting *Enterococcus gallinarum* [57], suggesting that reported plaque diameters often represent average values rather than strict uniformity. The presence of a faint halo around the plaques likely indicates the activity of lytic enzymes such as endolysins, holins, or depolymerases, which may diffuse into the surrounding bacterial lawn during lysis [58]. This interpretation was further supported by gene annotation analysis of *vB_EfaS_LOK1* genome.

Transmission electron microscopy (TEM) revealed that *vB_EfaS_LOK1* possesses an icosahedral capsid and a long, non-contractile tail, morphological features characteristic of bacteriophages formerly classified withing the *Siphoviridae* family. This structural profile is consistent with other *Enterococcus*- infecting phages, including 9183, 9184 [59], *IME-EF1* [9], *vB_EfaS_PHB08* [29], and *SFQ1* [60].

Phage host range is primarily determined by interactions between phage receptor-binding proteins and bacterial surface structures, as well as the presence of host defense mechanisms. In Gram-positive bacteria, adsorption often involves surface components such as peptidoglycan, murein, and teichoic acids [61]. While some *Enterococcus* specific phages display high host specificity [23,56], others exhibit broader tropism, infecting multiple strains or even different species [9,29,60,62,63]. *vB_EfaS_LOK1* demonstrated a narrow host range, which may be attributable either to the limited number of strains tested (eight *E. faecalis* and eleven *E. faecium*) or to the presence of bacterial resistance mechanisms, such as, receptor modification or masking, outer membrane vesicles, abortive infection systems, restriction-modification systems, or superinfection exclusion [64,65,66].

Phages generally tolerate neutral pH and a wide range of temperatures. *vB_EfaS_LOK1* remained viable across a slightly acidic to alkaline pH range (pH 4 to 11), and retained stability at temperatures up to 70 °C. These findings are consistent with the pH and temperature tolerance reported for other *E. faecalis* phages, including *vB_Efm_LG62* [67], *vB_EfaS-271* [23], *vB_EfaS-SRH2* [68], and *PHB8* [29], and may reflect the environmental versatility of the host bacterium. Moreover, the ability of *vB_EfaS_LOK1* to maintain lytic activity across a wide temperature range underscores its potential applicability under diverse physiological, environmental, and storage conditions [69].

One-step growth curve analysis revealed that *vB_EfaS_LOK1* has a short latent period and a 70 min burst period, producing approximately 72 plaque-forming units (PFU) per infected cell. While some phages exhibit shorter burst times (8–30 min) [23,63,68,70], others, such as *vB_EfaS_LOK1*, display longer replication cycles, with burst periods extending from 60 to 110 min [69,71,72]. Nonetheless, the median latent period and burst size for tailed phages generally fall within 40–60 min and 50–100 virions per infected cell, respectively [29]. Within this framework, the lytic performance of *vB_EfaS_LOK1* can be considered comparatively efficient.

Ideally, a repository of fully characterized phages would be available to support the formulation of tailored therapeutic cocktails. However, maintaining long-term phage viability during storage remains a significant challenge [34,73]. Our findings indicate that tryptic soy broth (TSB) supplemented with CaCl_2_ and MgCl_2_ (222 mg/L) was the most effective condition for preserving the viability of phage *vB_EfaS_LOK1*. These stabilizing effects align with previous studies highlighting the critical role of divalent cations in maintaining phage structural integrity [74,75]. In contrast, storage in SM buffer at 4 °C resulted in substantial viability loss, possibly due to salt precipitation at low temperatures or insufficient structural protection. Media enriched with proteins or polymers, which have demonstrated superior stabilizing capacity offer improved alternatives [34,74].

A limitation of our study was the relatively short duration of the storage evaluation, and the limited number of storage conditions tested. Future research should explore extended storage periods and a broader range of stabilizing formulations, including Ficoll, gelatin, or higher concentrations of Mg^2+^ or Ca^2+^. Lyophilization also warrants consideration, as it has been shown to preserve phage viability for 8 to 18 years while facilitating transport and long-term storage [73,75].

Phylogenomic and comparative analyses clearly placed *vB_EfaS_LOK1* within the ICTV-recognized genus *Efquatrovirus.* Notably, average nucleotide identity (ANI) analysis showed that *vB_EfaS_LOK1* shares 96.64% identity with *vB_OCPT_CCS4*, indicating that both belong to the same species-level group. However, this pair exhibited ANI values below 95% when compared to all ICTV-designated type species, strongly supporting the classification of *vB_EfaS_LOK1* and *vB_OCPT_CCS4* as members of a newly isolated bacteriophages species within the genus *Efquatrovirus*. This conclusion was further supported by intergenomic similarity values calculated using VIRIDIC (Figure S1, Supplementary Materials).

This classification is consistent with other *Enterococcus*-infecting phages that share conserved genomic architecture and virion morphology yet exhibit sufficient nucleotide-level divergence to warrant designation as distinct species. The recognition of *vB_EfaS_LOK1* as a putative newly isolated bacteriophage species underscores the ongoing genomic diversification of enterococcal phages and highlights the need for continued effort in phage isolation and characterization.

Comparative genomic analysis confirmed the strictly lytic nature of *vB_EfaS_LOK1*. Other *Enterococcus* phages, such as *vB_Efs8_KEN04* [71], *vB_EfaS_HEf13* [63], and *vB_EfKS5* [76], have likewise been reported to lack lysogeny-associated genes and other undesirable genetic elements. Similarly, *vB_EfaS_LOK1* harbors no genes associated with antimicrobial resistance or virulence. Its genome, approximately 41 kb in length, falls within the typical size range for *Enterococcus* phages, except for reduced-genome variants such as EFAP_1 (~21 kb) [77]. The GC content (34.8%) suggests evolutionary conservation and host-adaptive genomic stabilization under selective pressures.

Functionally, *vB_EfaS_LOK1* encodes key structural and replication-associated genes required for a canonical lytic replication cycle, including DNA polymerase, primase, helicase, and complete virion assembly proteins. The presence of lysis-associated genes, such as holins and endolysins, further supports its capacity for efficient host cell disruption and progeny phage release. Additionally, the annotation of multiple HNH endonucleases suggests potential roles in host DNA degradation or recombination, although these proteins may also represent mobile selfish elements rather than essential components of phage biology. Notably, termini analysis revealed that *vB_EfaS_LOK1* possesses a linear genome with cohesive ends, a feature consistent with other enterococcal phages such as EF4 [78], reinforcing the accuracy of its genomic characterization.

While our genomic analyses strongly support the classification of *vB_EfaS_LOK1* as a newly isolated bacteriophage species within the genus *Efquatrovirus*, it is important to recognize that viral taxonomy remains a dynamic and evolving field. Species demarcation is currently based on ICTV guidelines and available set of reference genomes, which continue to expand. Consequently, although both ANI and intergenomic similarity metrics consistently position *vB_EfaS_LOK1* and *vB_OCPT_CCS4* as a distinct lineage separate from all ICTV-recognized type species, future updates in phage taxonomy may further refine its classification.

The *findings* of this study establish *vB_EfaS_LOK1* as a potential candidate for therapeutic applications targeting multidrug-resistant *Enterococcus faecalis*. It is strictly lytic lifestyle, absence of virulence and antimicrobial resistance genes, and thoroughly characterized genome satisfy key safety and efficacy criteria for potential clinical use. Additionally, its stability across a broad range of pH and temperature conditions, coupled with efficient replication dynamics, underscores its robustness under diverse environmental and physiological settings.

Nevertheless, the relatively narrow host range observed for *vB_EfaS_LOK1* underscores the need for further investigations. Future research should focus on comprehensive host range profiling, along with in vitro and in vivo evaluations of efficacy and safety. Detailed analysis of receptor-binding interactions will be essential to elucidate the molecular basis of host specificity. Genetic engineering of receptor-binding proteins may offer a viable strategy to broaden host range and enhance therapeutic potential. Additionally, the development of optimized storage formulations and the incorporation of *vB_EfaS_LOK1* into well-defined synergistic phage cocktails could further improve its clinical applicability.

These findings contribute to the growing body of evidence supporting phage therapy as a viable alternative to conventional antibiotics, particularly in the context of the escalating global challenge of antimicrobial resistance.

## Data Availability

The original contributions presented in this study are included in the article/Supplementary Material. Further inquiries can be directed to the corresponding authors.

## Supplementary Materials

The following supporting information can be downloaded at https://www.mdpi.com/article/10.3390/microorganisms13102414/s1: Table S1. Antimicrobial susceptibility profiles of clinical strains used in the host range assessment of phage vB_EfaS_LOK1. Figure S1. Intergenomic Similarity Heatmap of *Enterococcus* Phages (VIRIDIC Analysis). This heatmap illustrates the pairwise intergenomic similarity (upper-right triangle, expressed as percentages) and the aligned genome fraction (lower-left triangle, indicated by color intensity) for 19 *Enterococcus* phages and *Staphylococcus* phage Twort. The phage of interest, *Enterococcus* phage *vB_EfaS_LOK1* (PV780768), is represented by contig00001. Phage *vB_EfaS_LOK1* shows a 97.6% intergenomic similarity with *Enterococcus* phage *vB_OCPT_CCS4* (ON113176), surpassing the 95% threshold for species demarcation and therefore classifying them as the same species. In contrast, excluding *vB_OCPT_CCS4*, the similarity between *vB_EfaS_LOK1* and all other *Enterococcus* phages remains below 80% (ranging from 70.5% to 79.6%). This pattern confirms its distinction from other species groups while still placing it within the same genus, as defined by the ≥70% threshold.

## Abbreviations

The following abbreviations are used in this manuscript:

ANOVA	Analysis of variance
CAUTIs	Catheter-associated urinary tract infections
CLSI	Clinical and Laboratory Standards Institute
GBDP	Genome-BLAST Distance Phylogeny
HAIs	Healthcare-associated infections
LB	Luria–Bertani
SM	Sodium–Magnesium
MDR	Multidrug-resistant
MEGA	Molecular Evolutionary Genetics Analysis
MOI	Multiplicity of infection
PDR	Pandrug-resistant
RHOVE	Hospital Epidemiological Surveillance Network
TEM	Transmission electron microscopy
TSA	Tryptic soy agar
TSB	Tryptic soy broth
PFU	Plaque-forming units per mL
VICTOR	Virus Classification and Tree Building Online Resource
XDR	Extensively drug-resistant
